# Analysis of the use and advantages of depth of anaesthesia monitoring in children undergoing tonsillar adenoid surgery

**DOI:** 10.4314/ahs.v25i1.46

**Published:** 2025-03

**Authors:** Tingting Sun, Hui Lin

**Affiliations:** Department of Anesthesiology, The First People's Hospital of Shuangliu District, Chengdu/West China (Airport) Hospital of Sichuan University, Chengdu, China

**Keywords:** Tonsil, Adenoid, Surgery, Anesthesia, Depth, Dexmedetomidine, Hemodynamics

## Abstract

**Background:**

To investigate the application and analysis of the advantages of depth of anesthesia monitoring in children undergoing tonsillar adenoid surgery.

**Methodology:**

Eighty tonsillar adenoid surgery patients were divided into a control group and an observation group. Observation group was given dexmedetomidine. Saline was given to the control group. Hemodynamics, perioperative indicators, stress reaction indicators, and adverse reactions were compared.

**Results:**

At t0, no statistically significant differences were found between the two groups for mean arterial pressure (MAP) (t =0.3789) or heart rater (HR) (t =0.0130). On t1, t2, t3 and t4, the mean arterial pressure (MAP) (tt1=4.5321, tt2=2.5818, tt3=5.0025, tt4=3.2068) and heart rater (HR) (tt1=6.64299, tt2=29.4580, tt3=15.5095, tt4=10.1461) were lower compared to the control group (P<0.05). Extubation time (t=49.9687) and respiratory recovery time (t=16.4542) of children in the observation group were shorter than those of the control group (t=20.1926), a statistical difference was found between the groups (P<0.05). Observation group adverse events were 0.44% lower than control group (x2=5.1647, P<0.05).

**Conclusion:**

During tonsillar adenoid surgery, dexmedetomidine hydrochloride was used to anesthetize children. The dosage of anesthetic drugs was adjusted according to the depth of anesthesia monitored. Despite its small effect on hemodynamic indexes, it delayed extubation, reduced organism stress, and facilitated respiratory management.

## Introduction

Clinical snoring in children is most commonly caused by adenoid hypertrophy and chronic tonsillitis^1^. If left untreated for a long time, these conditions can affect adjacent tissues, leading to problems with memory, concentration, and even mental retardation^2^. The standard treatment for adenoid hypertrophy and chronic tonsillitis is surgical excision^3^. However, due to the rich innervation of the oropharynx, this area of the body is highly responsive to surgical stimulation, and this may lead to haemodynamic disturbances and serious complications. Therefore, a high level of sedation and analgesia is required for surgical anaesthesia^4^. The choice of anaesthetic drugs and drug dosage is critical in order to minimize stress and improve the quality of anaesthesia without affecting the patient's awakening time or haemodynamics. Dexmedetomidine hydrochloride is a safe and effective option for paediatric surgical anaesthesia^5^, but its use in children with tonsillar adenoid hypertrophy under monitored depth of anaesthesia is still being explored.

## Patients and Methods

### Patients

Eighty children admitted for tonsillar adenoid surgery from April 2020 to December 2021 were selected for the study, and were divided into control and observation groups using the random number table method, with 40 cases in each group. In the control group, according to the American Society of Anesthesiologists (ASA) classification: 18 cases of class II and 22 cases of class I; height 85-121 cm (103.35±1.35) cm; weight 15-29 kg (22.65±0.24) kg; age 3-10 years (6.35±0.24) kg; 18 cases of female and 22 cases of male. In the observation group: according to ASA classification: 19 cases of grade II and 21 cases of grade I; height 84-122 cm (103.68±1.32) cm; weight 16-29 kg (22.59±0.23) kg; age 3-11 years (6.54±0.23) kg; 19 cases of females and 21 cases of males. There was no statistically significant difference between the basic information of the two groups of children (P>0.05). The study protocol was approved by the hospital ethics committee and the families of each child signed the informed consent form.

Inclusion criteria are as follows: (i) all patients present with frequent recurrent nasal congestion and runny nose, colds, combined with varying degrees of open-mouth breathing and sleep snoring. (ii) All patients have tonsillar hypertrophy >II with adenoid hypertrophy; (iii) All patients meet the indications for surgical excision. Exclusion criteria were as follows: (i) those with previous congenital heart disease or arrhythmia; (ii) those with excessive obesity; (iii) those with intellectual or psychiatric abnormalities; (iv) those with recent upper respiratory tract infections; (v) those with airway abnormalities; (vi) those with contraindications or allergies to intraoperative drugs or anaesthetic drugs; (vii) those who changed their intervention plan midway through the procedure. In regards to the informed consent process, we followed standard procedures for obtaining informed consent from the families of the children participating in our study. Prior to obtaining consent, the families were given a detailed explanation of the study, including the purpose, procedures, and potential risks and benefits. They were also given ample time to review and ask questions before signing the consent form. The consent form was written in clear and simple language to ensure understanding. We ensured that all participants had the right to withdraw from the study at any time without consequence. Additionally, we obtained ethical approval from the institutional review board before beginning the study to ensure that all ethical considerations were met.

### Methods

All patients were fasted for 4 h before surgery and intravenous access was established. 0.01 mg/kg of atropine (Guodianzhi H32020166, Jiangsu Lianshui Pharmaceutical Co., Ltd.) and 2 mg/kg of propofol (Guodianzhi H20040079, Sichuan Guorui Pharmaceutical Co., Ltd.) + 0.1 mg/kg of midazolam (Guodianzhi H20031037, Jiangsu Enhua Pharmaceutical Co., Ltd.) were administered intravenously 0.5 h before surgery. After falling asleep, the patient's body signs indicators (bispect ral index (BIS), Electrocardiogram (ECG), oxygen saturation(SpO2)) were monitored. Intraoperatively, glucose injection (4-2-1 method, 4 ml/kg for 10 kg and 2 ml/kg for 10 kg thereafter, then 1 ml/kg) was injected. In the observation group, 1 ug/kg of dexmedetomidine (Guodianzhi H20110085, Jiangsu Enhua Pharmaceutical Co., Ltd.) was pumped 10 min before induction, followed by 0.7 ug/kg-h. Intravenous administration of 0.3 ug/kg sufentanil (Guodianzhi H20054171, Yichang Renfu Pharmaceutical Co., Ltd.), 0.2 mg/kg of cis-trimethoprim Ltd.), 2 mg/kg of propofol (Guodianzhi H20040079, Sichuan Guorui Pharmaceutical Co.,Ltd.), 0.1 mg/kg of midazolam (Guodianzhi H20031037, Jiangsu Enhua Pharmaceutical Co. After 3 min of administration, tracheal intubation was performed. Pumped 0.2 ug/kg-min remifentanil (Guodianzhi H20143314, Jiangsu Enhua Pharmaceutical Co., Ltd.), 3 mg/kg-h propofol, 0.7 ug/kg-h dexmedetomidine. In the control group, saline was pumped in equal amounts 10 min before induction, and induction was the same as in the observation group, with 0.2 ug/kg·min remifentanil and 4-8 mg/kg·h propofol pumped in. We monitored the patient's BIS intraoperatively and gave 0.5 mg/kg propofol if the BIS was >55, and reduced the dosage of propofol or dexmedetomidine if the BIS was <45. The infusion of dexmedetomidine was stopped 10 min before surgery, and the infusion of remifentanil or propofol was stopped at the end of surgery. When he is awake and his tidal volume has recovered, perform aspiration and extubation.

### Observed indicators

The following indicators were observed: (i) Haemodynamic indicators, recording their mean arterial pressure (MAP) and heart rate (HR) before anaesthesia (t0), at intubation (t1), at the time of the opening device (t2), at the end of the operation (t3) and at the time of extubation (t4). (ii) Perioperative indicators, intraoperative propofol dosage, extubation time, and respiratory recovery time were recorded. (iii) Stress indicators, 4ml of their venous blood was collected and their cortisol (Cor) was measured by radioimmunoassay and their adrenaline indicators were measured by enzyme assay. (iv) Incidence of adverse reactions, the number of cases of postoperative delirium, nausea and vomiting, and postoperative agitation were recorded.

### Statistical analysis

Data were processed using Statistical Product and Service Solutions (SPSS) 20.0 statistical software (IBM, Armonk, NY, USA). Enumeration data were expressed as percentages or frequencies and compared using the chi-square test; measurement data were expressed as mean ± standard deviation (x̅ ± s), t-test was used for comparison. Differences were statistically significant when P < 0.05.

## Results

### Comparison of hemodynamic parameters between the two groups

There was no significant difference in MAP (t = 0.3789) which refers to mean arterial pressure and HR (t = 0.0130) which refers to heart rate indicators at t0 between the two groups (P > 0.05) (Table 1); at t1, t2, t3 and t4, MAP (tt1 = 4.5321, tt2 = 2.5818, tt3 = 5.0025, tt4 = 3.2068) and HR (tt1 = 6.4299, tt2 = 29.4580, tt3 = 15.5095, tt4 = 10.1461) in the observation group were lower than those in the control group, and the difference had statistical significance (P < 0.05) (Table 1).

**Table 1:** Comparison of hemodynamic parameters between the two groups [n, (%)]

Group	Number of subjects	T0 MAP (mmHg)	HR (beats/min)	T1 MAP (mmHg)	HR (beats/min)	T2 MAP (mmHg)	HR (beats/min)	T3 MAP (mmHg)	HR (beats/min)	T4 MAP (mmHg)	HR (beats/min)
Observation group	40	74.54±6.21	110.24±10.35	73.74±5.21	117.54±11.21	72.21±6.32	110.68±10.21	69.21±6.31	107.54±4.25	76.21±4.21	122.65±9.65
Control group	40	74.05±5.32	110.21±10.21	78.54±4.21	124.35±4.21	75.31±4.21	127.21±7.32	75.21±4.21	122.21±4.21	79.65±5.32	139.54±4.21
t	--	0.3789	0.0130	4.5321	6.4299	2.818	29.4580	5.0025	15.5095	3.2068	10.1461
P	--	0.7057	0.9896	0.0001	0.0006	0.0117	0.0001	0.0001	0.0001	0.0019	0.0001

### Comparison of indicators between the two groups

The intraoperative dosage in observation group was lower than that in control group (t = 20.1926). The extubation time (t = 49.9687) and respiratory recovery time (t = 16.4542) in control group had statistical significance (P < 0.05) (Table 2).

**Table 2:** Comparison of the indicators between the two groups (x̅ ± s)

Group	Number of subjects	Intraoperative dose (mg)	Time to extubation (min)	Resumption time (min)
Observation group	40	72.21 ± 10.21	29.68 ± 1.35	5.64 ± 1.02
Control group	40	118.65 ± 10.36	14.54 ± 1.36	9.98 ± 1.32
*t*	--	20.1926	49.9687	16.4542
*P*	--	0.0001	0.0001	0.0001

### Comparison of stress indicators between the two groups

Comparison of stress indicators at t0 and t1 in observation group (P > 0.05); at t2, t3 and t4, epinephrine and Cor in observation group were lower than that in control group, and the differences had statistical significance (P < 0.05) (Table 3).

**Table 3:** Comparison of stress parameters between the two groups (x̅ ± s)

Group	Number of subjects	T0 Epinephrine (ng/ml)	Cor(pg/ml)	T1 Epinephrine (ng/ml)	Cor(pg/ml)	T2 Epinephrine (ng/ml)	Cor(pg/ml)	T3 Epinephrine (ng/ml)	Cor(pg/ml)	T4 Epinephrine (ng/ml)	Cor(pg/ml)
Observation group	40	38.54 ± 4.21	149.65±11.21	49.87 ± 5.32	167.5 4±10.21	61.65 ± 7.21	182.54±11.21	78.12 ± 7.21	203.3 5±12.21	53.21 ± 4.21	191.21±12.32
Control group	40	40.15 ± 4.21 1.7	151.21±14.21 0.5	51.21 ± 4.65 1.1	168.65±10.23 0.9	77.65 ± 8.65 7.5	236.65±21.21 14.	85.24 ± 6.35 4.6	272.65±28.21 14.	68.21 ± 4.65 45.	219.65±13.21 9.9
t	--	1020.0	4510.5	9940.2	9350.6	6210.0	26510.0	8690.0	25840.0	14980.0	5770.0
P	--	912	872	340	285	001	001	001	001	001	001

### Comparison of the incidence of adverse events between the two groups

The incidence rate of adverse events in observation group (0.44%) was lower than that in control group (22.50%) (x2 = 5.1647, P < 0.05) (Table 4).

**Table 4:** Comparison of incidence rate of adverse events between the two groups [n,(%)]

Group	Number of subjects	Postoperative delirium	Nausea and vomiting	Postoperative agitation	Total Occurrence
Observation group	40	1 (2.50)	1 (2.50)	0 (0.00)	2 (0.44)
Control group	40	3 (7.50)	4(10.00)	2 (5.00)	9 (22.50)
X^2^	--				5.1647
P	--				0.0231

## Discussion

Because of its unique pharmacological properties, dexmedetomidine hydrochloride has gained application and acceptance in clinical anaesthesiology, particularly in adult patients^6^. In this study, it was used in children undergoing paediatric surgery. In children undergoing tonsillar adenoid surgery, the intense stimulation caused by intraoperative pharyngeal manipulation and tracheal intubation can increase blood pressure and heart rate. bradycardia and lower blood pressure. The slowing of heart rate caused by this drug is resistant to the increase in heart rate induced by surgical stimulation and tracheal intubation. The results of this study also showed that MAP and HR were lower in the observation group than in the control group at t1, t2, t3 and t4, and that epinephrine and Cor were lower in the observation group than in the control group at t2, t3 and t4, (P<0.05), also confirming that dexmedetomidine does not increase the haemodynamic response or the organism's stress response.

Tonsil adenoidectomy operations pose several risks to children, including postoperative bleeding, aspiration of secretions, and hypoxaemia. Prolonging the extraction time can help reduce the risk of bleeding and aspiration^7^, but it also requires careful management of anaesthesia to prevent hypoxaemia and respiratory depression. Dexmedetomidine is a useful anaesthetic agent in this context due to its dose-dependent analgesic, sedative, and anxiolytic effects, which can help reduce the neefor other anaesthetic drugs and minimize the risk of adverse events. The dexmedetomidine used in this study has a dose-dependent analgesic, sedative and anxiolytic effect, which reduces the dose of induction and maintenance anaesthetic drugs^8^.

This study also showed that the intraoperative propofol dosage was lower in the children in the observation group than in the control group, and the extubation time and respiratory recovery time were shorter than in the control group (P<0.05), indicating that the use of BIS to monitor the depth of anaesthesia during anaesthesia has positive implications in terms of controlling the amount of anaesthetic drugs administered. In addition, reducing the amount of anesthetic drugs administered also reduced the incidence of adverse events. The data from this study showed that the incidence of adverse events in the observation group was 0.44% lower than that in the control group at 22.50% (P<0.05), which is presumed to be directly related to the reduction in the amount of propofol administered. However, more specific mechanisms need to be analysed in depth.

## Conclusions

The use of dexmedetomidine hydrochloride in the anaesthesia of children undergoing tonsillar adenoid surgery, with the dosage of anaesthetic drugs adjusted according to the monitoring of the depth of anaesthesia, has very little effect on their haemodynamic indexes and reduces the incidence of organismal stress, postoperative adverse reactions, delays extubation and facilitates respiratory management.
